# A rapid high-performance semi-automated tool to measure total kidney volume from MRI in autosomal dominant polycystic kidney disease

**DOI:** 10.1007/s00330-018-5918-9

**Published:** 2019-01-21

**Authors:** Roslyn J. Simms, Trushali Doshi, Peter Metherall, Desmond Ryan, Peter Wright, Nicolas Gruel, Maatje D. A. van Gastel, Ron T. Gansevoort, Wendy Tindale, Albert C. M. Ong

**Affiliations:** 1 Kidney Genetics Group, Academic Unit of Nephrology, Department of Infection, Immunity and Cardiovascular Disease, University of Sheffield, Sheffield, UK; 2 Sheffield Kidney Institute, Sheffield Teaching Hospitals NHS Foundation Trust, Sheffield, UK; 3 Institute for in silico Medicine, University of Sheffield, Sheffield, UK; 4 Medical Imaging and Medical Physics, Sheffield Teaching Hospitals NHS Foundation Trust, Sheffield, UK; 5 Department of Nephrology, University Medical Center Groningen, Groningen, the Netherlands

**Keywords:** Polycystic kidney diseases, Autosomal dominant polycystic kidney disease, Magnetic resonance imaging, Kidneys

## Abstract

**Objectives:**

To develop a high-performance, rapid semi-automated method (Sheffield TKV Tool) for measuring total kidney volume (TKV) from magnetic resonance images (MRI) in patients with autosomal dominant polycystic kidney disease (ADPKD).

**Methods:**

TKV was initially measured in 61 patients with ADPKD using the Sheffield TKV Tool and its performance compared to manual segmentation and other published methods (ellipsoidal, mid-slice, MIROS). It was then validated using an external dataset of MRI scans from 65 patients with ADPKD.

**Results:**

Sixty-one patients (mean age 45 ± 14 years, baseline eGFR 76 ± 32 ml/min/1.73 m^2^) with ADPKD had a wide range of TKV (258–3680 ml) measured manually. The Sheffield TKV Tool was highly accurate (mean volume error 0.5 ± 5.3% for right kidney, − 0.7 ± 5.5% for left kidney), reproducible (intra-operator variability − 0.2 ± 1.3%; inter-operator variability 1.1 ± 2.9%) and outperformed published methods. It took less than 6 min to execute and performed consistently with high accuracy in an external MRI dataset of T2-weighted sequences with TKV acquired using three different scanners and measured using a different segmentation methodology (mean volume error was 3.45 ± 3.96%, *n* = 65).

**Conclusions:**

The Sheffield TKV Tool is operator friendly, requiring minimal user interaction to rapidly, accurately and reproducibly measure TKV in this, the largest reported unselected European patient cohort with ADPKD. It is more accurate than estimating equations and its accuracy is maintained at larger kidney volumes than previously reported with other semi-automated methods. It is free to use, can run as an independent executable and will accelerate the application of TKV as a prognostic biomarker for ADPKD into clinical practice.

**Key Points:**

• *This new semi-automated method (Sheffield TKV Tool) to measure total kidney volume (TKV) will facilitate the routine clinical assessment of patients with ADPKD.*

• *Measuring TKV manually is time consuming and laborious.*

• *TKV is a prognostic indicator in ADPKD and the only imaging biomarker approved by the FDA and EMA.*

**Electronic supplementary material:**

The online version of this article (10.1007/s00330-018-5918-9) contains supplementary material, which is available to authorized users.

## Introduction

Autosomal dominant polycystic kidney disease (ADPKD) is the most common inherited kidney disease and fourth leading cause of end stage renal failure (ESRF) worldwide [1, 2]. It is characterised by the gradual progressive development and growth of renal cysts which result in increased total kidney volume (TKV).

Changes in estimated glomerular filtration rate (eGFR) are conventionally used to measure loss of kidney function. In ADPKD, however, eGFR does not change until the later stages of disease due to compensatory glomerular hyperfiltration [3] thus limiting its use to late disease [4]. At earlier stages of disease, increases in TKV are detectable before decreases in eGFR [5]. A single baseline TKV measurement in combination with age and eGFR (Mayo Imaging Classification) has been shown to accurately predict future decline in kidney function [6]. TKV has been approved by both the US Food and Drug Administration (FDA) and the European Medicines Agency (EMA) as a prognostic biomarker for disease progression in ADPKD to facilitate the enrichment of patients at a higher risk of rapid progression in future clinical trials and is currently the only approved imaging biomarker [4]. In addition, tolvaptan [7] has been licenced for use in ADPKD patients in Europe with ‘evidence of rapid disease progression’. Guidance from the ERA-EDTA recommends the use of TKV to select higher risk patients for treatment [8].

The current gold standard method for measuring TKV from MRI involves manual tracing of the kidney boundary on each MRI slice using dedicated software and summing the product of area measurements and slice thickness [9]. This is time consuming and subject to intra- and inter-operator variability errors. The alternative method of stereology involves specialised software which creates a grid over the kidney [10]. There is a clear need to develop more rapid and accurate methods for measuring TKV to facilitate its wider adoption into clinical use.

Several semi-automated methods and estimating equations have been developed to address the challenge of measuring TKV in ADPKD [11] (summarised in Table S1). Fully automatic methods to estimate TKV have also been reported [12–14] although they require a good training dataset to include severely cystic kidneys due to the associated geometric and anatomical variability. A recent informative review [15] discusses these different techniques and highlights the need to translate them into clinical practice to inform disease progression and treatment decisions. Furthermore, a recent comparison of various methods of measuring TKV [16] in ADPKD concluded that, compared to manual segmentation, existing methods are insufficiently accurate.

In this paper, we describe the development and validation of a rapid, high-performance semi-automated method (*Sheffield TKV Tool*) for measuring TKV in a representative group of patients with ADPKD and a wide range of TKV.

## Materials and methods

### Study population

Sixty-one patients with ADPKD and stage 1–3 chronic kidney disease (CKD) attending a specialist PKD clinic at Sheffield Kidney Institute consented to an unenhanced abdominal MRI for measurement of TKV. Renal function (eGFR [17]) was measured at baseline (within 1 month of the MRI) and the most recent follow-up result was recorded (2.00 ± 0.52 years; 0.07–2.72 years). The study was approved by a research ethics committee (13/YH/026).

### MRI acquisition

Kidney MRI scans were coronal true fast imaging with steady-state free precession (TRUFI) T2-weighted sequences (Siemens Avanto 1.5-T scanner) with the following parameters: 4 mm slice thickness, 0 mm slice gap, 2 ms echo time, 3.99 ms repetition time, 60° flip angle, 0.68 × 0.68 mm in plane resolution and 512 × 512 acquisition matrix. The TRUFI sequence acquisition time was 138 s. This imaging sequence was selected because its images enabled the clearest delineation of the kidney and associated cysts from neighbouring tissues.

### Sheffield TKV Tool development

**Fig. 1:**
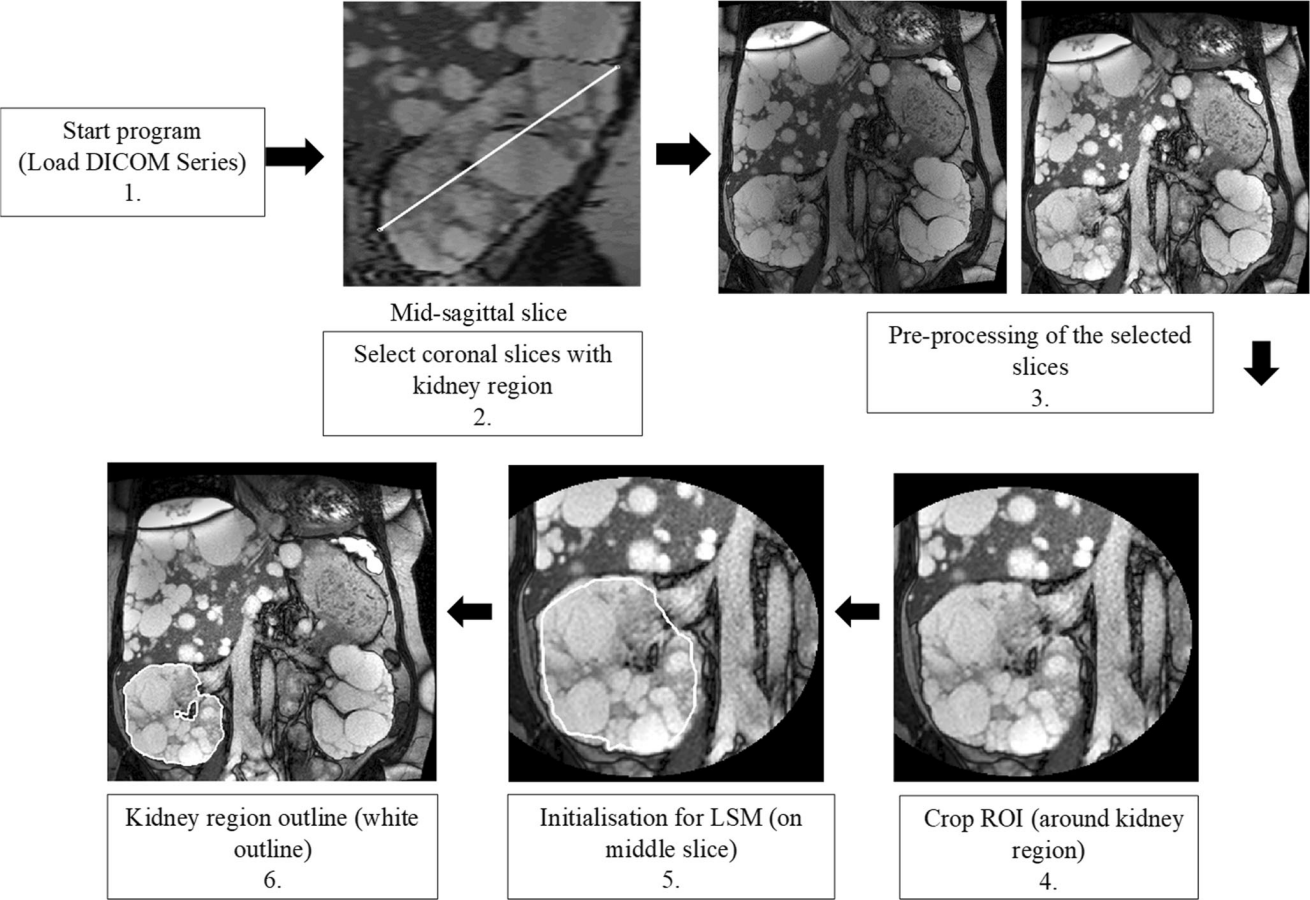
Flow chart of Sheffield TKV Tool. **1.** The tool initially loads DICOM (T2 TRUFI) series. **2.** The user selects the approximate sagittal mid-slice and identifies two points to define the kidney edge which allows selection of coronal slices that contain the kidney. **3.** The selected slices are pre-processed to remove motion artefact, intra- and inter-slice intensity variations. Step 3 shows MRI slices before and after pre-processing respectively. **4.** The user previews the cropped image to check the entire kidney is included within the defined region and **5.** Level set method is manually initialised near the kidney region boundary (white outline) only on the mid-coronal slice, **6.** The final kidney region outline (white outline) is obtained based on a hybrid level set method

The Sheffield TKV Tool was implemented using a MATLAB 2016b (MathWorks) framework. The right or left kidney was segmented individually from coronal MRI slices using image processing techniques (Fig. 1). Coronal kidney region slices were selected using mid-sagittal plane (Fig. 1: Step 2). Prior to segmentation, pre-processing steps were applied to reduce motion artefact and intra- and inter-slice intensity variations [18] from the selected coronal slices (Fig. 1: Step 3). Motion artefact was corrected using affine registration (rotation and translation) between slices. To correct intra-slice intensity (bias field) variation, entropy minimisation technique was employed [19]. To correct inter-slice intensity variation, a technique used by MIROS [18] was employed which minimised the mean square error of a threshold-based segmentation (with the number of adjacent voxels greater than half-slice maximum value) by finding the best-fit linear multiplier to allow tissue signal homogeneity between slices.

On the cropped region of interest (Fig. 1: Step 4), the user-defined contour (Fig. 1: Step 5) was propagated using hybrid level set method (LSM) [20] that utilise edge (gradient) as well as regional statistics to obtain final segmentation boundary. The energy function *E*(*ϕ*) used in hybrid LSM [20] is given as:

$$ E\left(\phi \right)=-a\underset{\varOmega }{\int}\left(I-u\right)H\left(\phi \right) d\varOmega +\beta \underset{\varOmega }{\int }g\mid \nabla H\left(\phi \right)\mid d\varOmega $$

where *ϕ* is the level set function. *I* is the image to be segmented; *H(ϕ)* is smooth Heaviside function which considers area around contour; *g* is image edge (boundary) map where contour should be attracted and is set to be $g=\frac{1}{1-c{\left|\nabla I\right|}^2}$ with *c* controlling the slope. *Ω* is the image domain and *α* and *β* are predefined weights to balance two terms. The first term on the right-hand side of the equation defines that region to be segmented should have intensity greater than *μ* which is set to 50. Parameters *α* and *β* are set to 0.01 and 100, respectively. Evolving contour (level set function) is stopped after 100 iterations to obtain expected kidney region outline (Fig. 1: Step 6). These parameters were determined experimentally from a random dataset of 10. The final contour obtained is not highly sensitive to the choice of parameters. After segmentation, kidney volume (KV) was calculated by summing the product of areas of the kidney region and slice thickness. The Sheffield TKV Tool was applied separately for the right (R) and left (L) kidneys to enable errors specific to either side to be identified quickly. The tool was developed and optimised using 10 random cases from the patient cohort (training set) and internally validated on the remaining (51) patient images.

### Mayo risk classification

We classified patients in the development cohort into class 1 (typical (bilateral, diffuse)) or class 2 (atypical (unilateral, segmental, asymmetric cystic disease) based on their kidney morphology on MRI as defined in the Mayo Imaging classification [6]. Class 1 patients were further subdivided into categories 1A–1E, which has been shown to correlate with the rate of disease progression measured by eGFR change [6].

### TKV measurements

To obtain reference TKV measurements, the gold standard method of manual segmentation was performed using MIM Maestro on T2 TRUFI coronal MR images of all 61 patients by an experienced image analyst (*A*). Analyst *A* was blinded to the development and TKV measurements of the Sheffield TKV Tool. Consistent with standard methods of manually measuring TKV [9, 18], blood vessels in the kidney and hilum (structures including ureter, blood vessels and nerves entering each kidney) were excluded [21]. A second image analyst (*B*) used the Sheffield TKV Tool (*B*^SheffieldTKVTool1^) to measure the right (R) and left (L) kidney volumes (KV) separately and compared its performance to that of the mid-slice method [6, 22] and MIROS tool [18] in all 61 patients and used the ellipsoid formula on 51 (typical, class 1) patients. Images from class 2 patients were excluded from TKV analysis using the ellipsoid formula because the developers [6] advise it is not reliable for use in atypical ADPKD since these patients do not have an ellipsoid kidney shape.

TKV was obtained by summing right and left KV. To apply the MIROS tool, open-source code was obtained from https://gitlab.com/Philbrick/rilcontour and re-written in MATLAB. The MIROS tool was developed for HASTE sequences. The MIROS algorithm parameters were tuned on a training set of 10 random representative (TRUFI image) cases that were used to optimise the parameters of the MIROS Tool (described in detail in their methods [18]), particularly parameters alpha (a constant to adjust the gradient strength) was altered from 1e5 to 1e4 and sigma (standard deviation of the population) was reduced from 3 to 1.

To assess the inter-operator variability of manual segmentation, analyst *B* manually (*B*^manual1^) measured TKV for 40 kidneys from a representative subset of 20 patients (TKV 258–3680 ml) for comparison with TKV already measured in this dataset by analyst *A*^manual^. Analyst *B* repeated the manual segmentation on the same dataset after 1 month (*B*^manual2^) to assess the intra-operator variability.

To assess the inter-operator variability of the Sheffield TKV Tool, analyst *A* measured TKV for the same dataset of 20 patients (*A*^SheffieldTKVTool^). The intra-operator variability of the Sheffield TKV Tool was assessed by analyst *B* on two occasions (*B*^SheffieldTKVTool1^, *B*^SheffieldTKVTool2^) separated by 1 month.

### Validation of the Sheffield TKV Tool

T2-weighted MRI renal images of 65 ADPKD patients who participated in the DIPAK-1 study [23] were acquired using one of three 1.5-T scanners (GE Medical Systems (16), Siemens (37) and Philips Healthcare (12)). De-identified DICOM image data from the DIPAK-1 study was transferred to Groningen Medical Center and converted to the NIFTI file format by the dcm2nii software. The images had a reconstructed matrix size of at least 256 × 256 × Z (with Z large enough to cover the full extent of the kidneys within the imaged volume). Image voxel sizes were most commonly on the order of 1.5 mm in-plane with 4-mm slice thickness and spacing between slices. The Medical Ethics Committee of University Medical Center Groningen approved the trial protocol that was conducted in accordance with the International Conference of Harmonization Good Clinical Practice Guidelines and in adherence to the ethics principles that have their origin in the Declaration of Helsinki. All the patients gave written informed consent.

Kidney boundaries were manually traced using commercially available software AnalyzeDirect 11.0 (AnalyzeDirect Inc.) and kidney volumes were calculated from the set of contiguous images by summing the products of the area measurements within the kidney boundaries and slice thickness. Non-renal parenchyma, e.g. the renal hilum, was excluded from measurement. Importantly, all measurements were performed by readers blinded for patient number and previous TKV measurements [9]. Separate KV for the left and right kidneys was determined using MATLAB software to separate the measured TKV. The Sheffield TKV Tool was used (analyst *B*) to measure TKV on this dataset in a blinded manner and its performance relative to the reference manual segmentation values was then assessed.

### Statistical analyses

Baseline demographics are reported as mean ± standard deviation (SD). Agreement was assessed using Bland-Altman (BA) analyses to determine the mean difference between TKV for the various methods. Both actual and percentage (%) difference in volume were evaluated. For development of the Sheffield TKV Tool, sample size was calculated for BA agreement assessment [24] using the level of significance *ρ* (type I error), a power value (type II error), expected mean, SD and maximum acceptable percentage (%) volume difference between reference and TKV Tool measurements [24]. Based on published literature [12, 18], the expected mean (bias in BA plot) was 2%, the expected SD (precision in BA plot) 5% and the maximum allowed difference 15% (greater than mean + 2SD) [24]. Thus, for *ρ* of 0.05, a power of 0.80, the minimum required number of TKV measurement pairs was 60. Bias (mean) obtained from different methods was assessed using paired sample *t* test.

Spatial overlap between segmentation outlines was determined using the dice similarity coefficient (DSC) [25]. A DSC value of 1 implies complete overlap while a value of 0 implies no overlap. Inter- and intra-operator variability were also assessed by coefficient of variation (CoV) [26].

## Results

### Characteristics of the development cohort

**Fig. 2:**
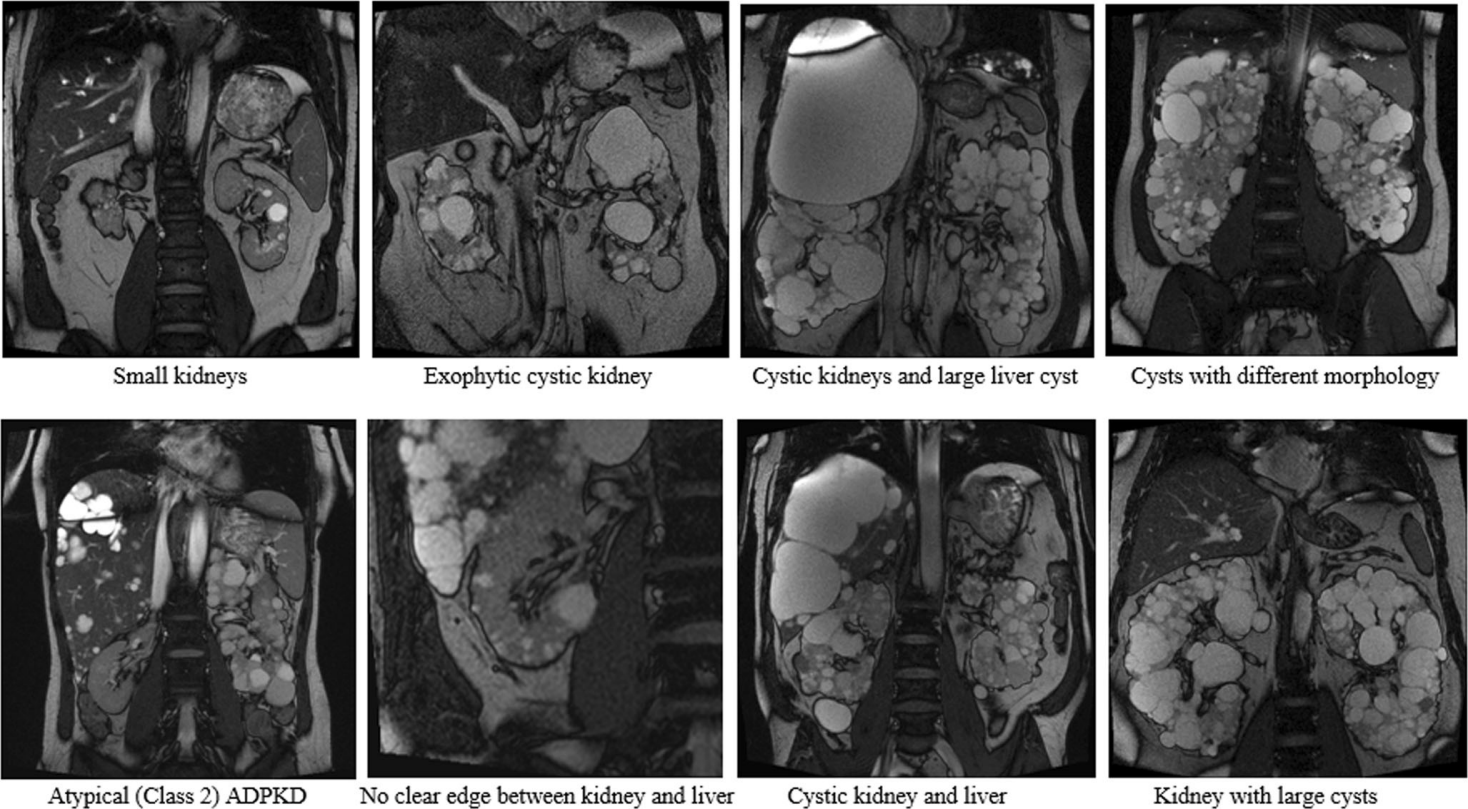
Representative ADPKD kidney MR images used to test Sheffield TKV Tool

Sixty-one ADPKD patients (32 female, 29 male) with a mean age of 45 ± 14 (20–77) years and eGFR of 76 ± 32 ml/min/1.73 m^2^ (33–175 ml/min/1.73 m^2^) participated in this study. They represented a wide spectrum of disease with gold standard TKV ranging (mean ± SD) between 258 and 3680 ml (1167 ± 798 ml). Their kidneys had variable morphology (shape, size and heterogeneous cysts) (Fig. 2) and 42 (69%) patients had liver cysts. Based on the Mayo imaging classification [6], 51 patients were categorised as class 1 (typical) and 10 patients as class 2 (atypical disease). Class 1 patients were further subdivided into five prognostic groups (1A–1E) [6].

### Performance of the Sheffield TKV Tool

Table 1 summarises the performance of the Sheffield TKV Tool, the ellipsoid, mid-slice and MIROS methods compared to the reference gold standard manual method in all 61 patients (122 kidneys). The mean TKV of 1153 ± 786 ml (258–3737 ml), measured by the Sheffield TKV Tool, was close to manually measured TKV (1167 ± 798 ml; 258–3680 ml), whereas the ellipsoid (1238 ± 742 ml; 261–3437 ml), mid-slice (1196 ± 827 ml; 276–4082 ml) and MIROS (1182 ± 821 ml; 261–3780 ml) methods overestimated TKV.

**Table 1:** Accuracy and precision of different semi-automated methods of measuring or estimating KV compared to manual segmentation

		Volume (ml) (mean ± SD)	% volume difference (mean ± SD)	Raw volume difference (mean ± SD)
Right KV	Manual (reference)	563 ± 400	–	–
	Ellipsoid method*	568 ± 394	4.5 ± 19.7	11.0 ± 129.4
	Mid-slice method	568 ± 405	1.9 ± 11.1	4.1 ± 72.6
	Sheffield TKV Tool	561 ± 392	0.5 ± 5.3	− 2.8 ± 25.3
Left KV	Manual (reference)	597 ± 417	–	–
	Ellipsoid method*	576 ± 378	1.7 ± 17.6	− 21.9 ± 162.9
	Mid-slice method	629 ± 452	6.1 ± 12.4	31.8 ± 79.0
	Sheffield TKV Tool	592 ± 419	− 0.7 ± 5.5	− 5.1 ± 29.7
TKV	Manual (reference)	1167 ± 798	–	–
	Ellipsoid method*	1238 ± 742	3.1 ± 14.1	− 10.6 ± 223.76
	Mid-slice method	1196 ± 827	3.8 ± 9.2	35.9 ± 104.9
	MIROS Tool	1182 ± 821	1.4 ± 5.1	21.7 ± 60.8
	Sheffield TKV Tool	1153 ± 786	− 0.3 ± 3.8	− 7.9 ± 41.8

Results are shown for all 61 patients (122 kidneys). Negative values indicate underestimation of KV compared to manual segmentation. Various methods were tested on images of kidneys with manual volumes (analyst *A*) as reference. The Sheffield TKV Tool was more accurate and precise compared to the other methods with no bias for either the left or right kidneys

*SD* standard deviation, *KV* kidney volume, *TKV* total kidney volume

*Results for Ellipsoid method is shown only for class 1 (typical) patients (51 patients)

For MIROS, no separate volumes were obtained for the left and left kidneys, thus results are reported for total kidney volume (TKV)

**Fig. 3:**
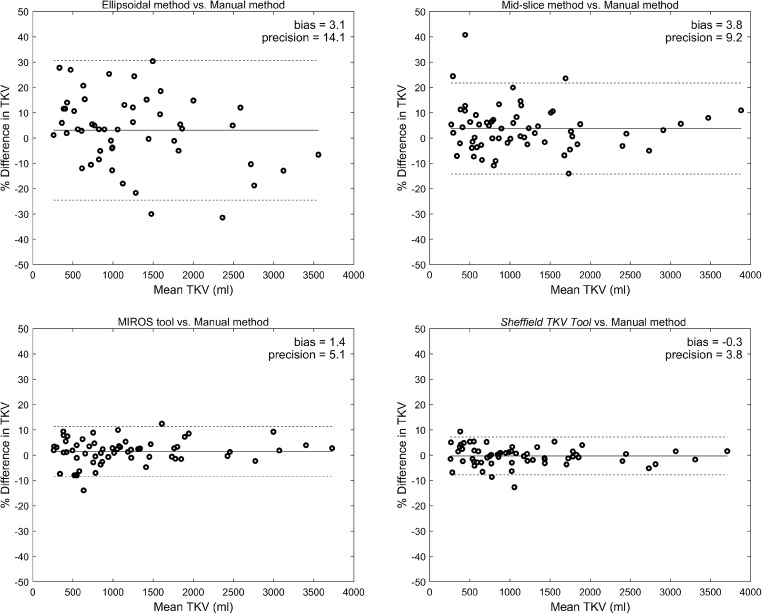
Bland–Altman analysis of different methods (ellipsoid, mid-slice, MIROS and Sheffield TKV Tool) to measure TKV compared to the reference manual method. Bland–Altman plots (bold line, mean; dashed lines, 95% confidence intervals) comparing the percentage (%) volume difference of each method to the reference manual method to measure TKV in 61 patients. The Sheffield TKV Tool demonstrates higher accuracy and precision compared to all other methods

In terms of volume error (Table 1, Fig. 3), the Sheffield TKV Tool performed more accurately and with greater precision with a mean TKV difference of − 0.3 ± 3.8% compared to the ellipsoid (3.1 ± 14.1%), mid-slice (3.8 ± 9.2%) and MIROS (1.4 ± 5.1%) methods. Paired sample *t* test however showed no statistically significant difference (*ρ* (2-tailed) = 0.008) between bias obtained using the Sheffield TKV Tool and MIROS method. The Sheffield TKV Tool demonstrated no particular bias (± ≤ 0.5%) and had a narrower 95% confidence interval. In contrast, the estimating equations were less reliable with a positive bias (overestimation of TKV) and more variable results (wider 95% confidence intervals).

Figure S1 shows the high agreement (0.89 ± 0.06 (RKV) and 0.90 ± 0.04 (LKV) of the DSC for the Sheffield TKV Tool compared to the manual method. There were high inter- (− 0.5 ± 3.5%, CoV 2.3) and intra- (0.5 ± 2.2%, CoV 1.6) operator reproducibility for manual TKV measurements. Inter- (1.1 ± 2.9%, CoV 2.2) and intra- (− 0.2 ± 1.3%, CoV 0.8) operator reproducibility for the Sheffield TKV Tool were higher than those for manual measurements (Table 2).

**Table 2:** Intra- and inter-operator variability to assess reproducibility and precision of the Sheffield TKV Tool

	Comparison		% volume difference (mean ± SD)	Raw volume difference (ml) (mean ± SD)	CoV (coefficient of variation) (%)
Right KV	Manual segmentation	Intra-operator (*B*^manual1^ vs. *B*^manual2^)	1.1 ± 2.6	5.4 ± 10.6	1.9
		Inter-operator (*A*^manual^ vs. *B*^manual1^)	0.9 ± 3.4	7.5 ± 12.5	2.4
	Sheffield TKV Tool	Intra-operator (*B*^SheffieldTKVTool1^ vs. *B*^SheffieldTKVTool2^)	− 0.1 ± 1.3	1.3 ± 4.2	1.0
		Inter-operator (*A*^SheffieldTKVTool^ vs. *B*^SheffieldTKVTool1^)	1.5 ± 4.6	3.5 ± 22.1	3.4
Left KV	Manual segmentation	Intra-operator (*B*^manual1^ vs. *B*^manual2^)	0.1 ± 2.2	1.0 ± 11.0	1.5
		Inter-operator (*A*^manual^ vs. *B*^manual1^)	0.2 ± 3.6	0.4 ± 14.7	2.5
	Sheffield TKV Tool	Intra-operator (*B*^SheffieldTKVTool1^ vs. *B*^SheffieldTKVTool2^)	−0.3 ± 1.3	− 1.0 ± 6.9	0.9
		Inter-operator (*A*^SheffieldTKVTool^ vs. *B*^SheffieldTKVTool1^)	1.0 ± 3.1	7.4 ± 17.6	2.3

Results obtained from a subset of 20 patients (40 kidneys). *KV* kidney volume, *SD* standard deviation

### Validation of the Sheffield TKV Tool in an external dataset

The MRI of 65 patients (25 female, 40 male) with ADPKD, mean age 50 ± 8 (26–61) years and eGFR of 52 ± 13 (33–78) ml/min/1.73 m^2^ with a representative spectrum of disease with gold standard TKV ranging (mean ± SD) between 400 and 7431 ml (2408 ± 1806 ml) were used. These patients also had more severe associated polycystic liver disease. Table 3 summarises the performance of the Sheffield TKV Tool compared to the reference gold standard manual method (AnalyzeDirect) in all 65 patients (130 kidneys). The mean TKV of 2344 ± 1806 ml, measured by the Sheffield TKV Tool, was close to the manually measured TKV (2408 ± 1806 ml).

**Table 3:** Validation: accuracy and precision of *Sheffield TKV Tool* compared to manual segmentation using external dataset

		Volume (ml) (mean ± SD)	% volume difference (mean ± SD)	Raw volume difference(mean ± SD)
Right KV	Manual (reference)	1149 ± 871	–	–
	Sheffield TKV Tool	1109 ± 862	3.91 ± 5.24	40.35 ± 60.38
Left KV	Manual (reference)	1259 ± 966	–	–
	Sheffield TKV Tool	1235 ± 981	3.14 ± 4.95	23.46 ± 99.42
TKV	Manual (reference)	2408 ± 1806	–	–
	Sheffield TKV Tool	2344 ± 1806	3.45 ± 3.96	63.81 ± 142.81

The reference TKV was provided by manual TKV measurements at University of Groningen using AnalyzeDirect 11.0 software (Spithoven Est TKV AJKD 2015). *SD* standard deviation, *KV* kidney volume, *TKV* total kidney volume

**Fig. 4:**
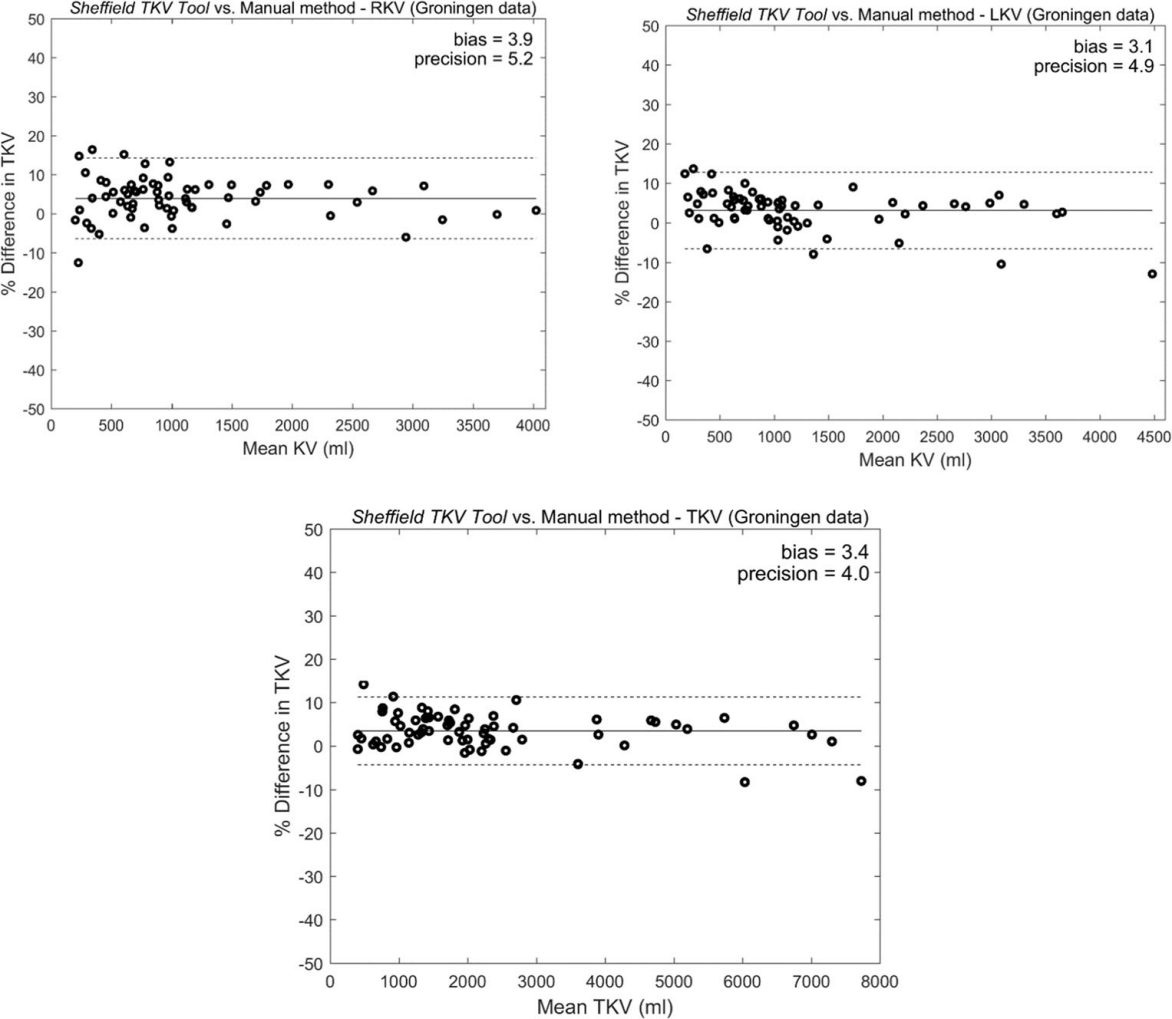
Bland–Altman analysis of Sheffield TKV Tool to measure TKV compared to the reference manual method for external (Groningen) dataset. **a** Right kidney volume. **b** Left kidney volume. **c** Total kidney volume. Bland–Altman plots (bold line, mean; dashed lines, 95% confidence intervals) comparing the percentage (%) volume difference of tool to the reference manual method to measure TKV in 65 patients

The mean volume error (Table 3, Fig. 4c) between the Sheffield TKV Tool and the manual reference for the external dataset was 3.45 ± 3.96%. This overall positive mean (bias) difference indicates the manual volume was greater than the value obtained by the tool. We attributed this mainly to the method of manual segmentation (AnalyzeDirect), which leaves a slight border around the kidney. The higher resolution of the internal dataset (pixel spacing 0.68 mm) compared to the external dataset (pixel spacing 1.5 mm) and the use of different scanners could also have contributed to this difference. Finally, the training dataset to determine values of parameters used in level set function was obtained from the internal dataset (61 patients) and was not optimised for the external dataset. Nonetheless, standard deviation (precision) values were comparable to the initial results obtained during development of the tool (Table 1). The higher mean volume error for right KV is likely to be secondary to the low contrast between the right kidney and liver, which was often very cystic. This difference was more prominent in the external dataset (Table 3) where the kidneys were clearly larger (twice the volume of the internal dataset) representing patients with later-stage disease consistent with their recorded age and renal function.

### Time taken to measure TKV

The average time taken to measure TKV by manual segmentation was 44 ± 18 min. In comparison, the Sheffield TKV Tool took 5.6 ± 1.5 min on the Sheffield cohort and 5 ± 3 min on the external validation cohort. The mean time to perform ellipsoid, mid-slice and MIROS methods was 4.5 ± 0.6, 3.2 ± 0.8 and 6.5 ± 2.2 min, respectively.

Table 4 shows the number of misclassified patients assigned to Mayo imaging classes (1A–1E) based on TKV calculated using various methods (manual, ellipsoid, mid-slice, MIROS and Sheffield TKV Tool). Compared to the manual method, the Sheffield TKV Tool misclassified 2 patients from class 1C to 1B and 1 patient from class1A to 1B. However, these two patients were misassigned from class 1C to 1B and class 1A to 1B by all four methods: in this case, the value for manual HtTKV was borderline between class 1B and 1C (age 51 years, HtTKV 678 ml) or between class 1B and 1A (age 36 years, HtTKV 256 ml) (please refer [6] Supp Table S2). In the third patient, the Sheffield TKV Tool significantly undersegmented the kidney region due to the presence of large cysts, a current limitation (see later). Overall, class assignment based on the Sheffield TKV Tool was comparable to the mid-Slice and MIROS methods and performed better than the ellipsoid method which misclassified 8 patients in total, 6 between classes 1B and 1C (Table 4).

**Table 4:** Number of class 1 (A–E) ADPKD (out of 51) patients [6] misclassified based on TKV measured using various TKV measurement tools. Assignment by manual TKV measurements was used as reference

TKV measurements methods	Class 1 misclassification					
	A to B	B to A	B to C	C to B	C to D	Total
Ellipsoid method	1	0	3	3	1	8
Mid-slice method	1	0	0	1	1	3
MIROS tool	1	1	0	1	0	3
Sheffield TKV Tool	1	0	0	2	0	3

## Discussion

We report a new semi-automated method (Sheffield TKV Tool) to measure TKV from MRI in ADPKD. The tool can run independently as a self-contained package and requires minimal user interaction to define a kidney outline from a coronal T2-weighted slice. Compared to manual segmentation, it performed with high accuracy in an unselected group of patients with a wider spectrum of disease than previously reported, as represented by kidneys with highly variable morphology, cyst burden, intensity distribution and extensive range of TKV (range 258–7431 ml). Importantly, it performed with high precision with no bias in measurements of the right or left kidneys, high agreement (mean DSC 0.90 ± 0.05, TKV difference − 0.3 ± 3.8%) and reproducibility (1.1 ± 2.9%) compared to the manual method. Validation in a representative external group of 65 patients with ADPKD confirmed good performance (mean volume error 3.45 ± 3.96%) with the positive bias caused by the method of manual segmentation which includes a slight border around the kidney.

A direct comparison between the Sheffield TKV Tool and two estimation methods (ellipsoid and mid-slice) in the same patients showed that it was more accurate and precise than either. Unlike the estimation methods, it also clearly outlined the kidney boundaries: these could be used as a precursor for the segmentation of renal cysts [27]. The Sheffield TKV Tool also performed as well as the MIROS [18] method in terms of precision and accuracy (Table 1). MIROS requires more manual interaction for larger kidneys and will therefore likely require more time in higher risk patients (Mayo class 1 C–E) since the user must draw a polygon in between slices to initiate kidney segmentation, unlike the Sheffield TKV Tool where manual interaction is independent of kidney size.

The Sheffield TKV Tool also performed better (TKV difference − 0.3 ± 3.8%) when compared to other published semi-automated methods [12, 28] (Table S1). Turco et al [28] reported a greater volume difference (− 1.3 ± 3.9%) in 30 patients despite a smaller TKV range (693–2029 ml). Kim et al reported a larger volume error of 4.2 ± 16.8% in 30 patients for training and 30 for validation and a smaller volume range of 177–2634 ml with their automatic method [12]. Although no manual interaction is required when measuring TKV with their technique, large volume errors (≥ 40% in 4 patients) resulted in the subsequent need for manual verification after segmentation.

In terms of efficiency, the reduced time required for the Sheffield TKV Tool would enable 8–10 TKV measurements to be performed in the time taken for a single manual TKV measurement. It performed particularly well on larger kidneys and in a wider range of kidney volumes (258–7431 ml) than previously reported (largest 2837 ml) [18, 28].

Liver cysts can cause considerable challenges when measuring TKV because of the close proximity of the liver with the right kidney and less often the left kidney (with much enlarged polycystic livers), since the distribution of cysts between the two neighbouring organs can be indistinguishable. However, in most cases, the Sheffield TKV Tool was able to distinguish between liver and kidney cysts even when the visual boundaries appeared vague. There was no influence of imaging classification on the performance of the Sheffield TKV Tool: it performed equally well in class 1 and class 2 patients. This is a considerable advantage since no patients requiring TKV measurements need to be excluded.

The current limitations of the Sheffield TKV Tool are a slight undersegmentation and measurement of TKV in kidneys associated with exophytic cysts or oversegmentation associated with large blood vessels especially when the kidney regions are small (Fig. S1j). It has been developed for use on T2-weighted MR sequences and has not been validated on T1-weighted images. However, a recent comparison of T1- or T2-weighted images for measuring TKV has reported that T2-weighted images were frequently of better quality to enable TKV measurements and were associated with improved reproducibility with lower intra- and inter-reader variability [29].

It has not yet been tested for serial measurements of TKV measurements to monitor natural history or response to treatment. A future goal is therefore to apply image registration techniques for this purpose [30]. Finally, the misclassification of 2 patients to a lower risk class (1C to 1B) based on TKV suggests that in cases with borderline TKV values between classes or with atypical outlines leading to undersegmentation, manual reanalysis may be required [6].

In summary, the accuracy, reproducibility and rapidity of the Sheffield TKV Tool highlight its potential for wider adoption to measure TKV as a prognostic marker routinely in patients with ADPKD.

## Electronic supplementary material

ESM 1 (DOCX 529 kb)

## Abbreviations and acronyms (non-commonly used)

CoV: Coefficient of variation
DSC: Dice similarity coefficient
HtTKV: Height-adjusted total kidney volume
LKV: Left kidney volume
LSM: Level set method
MIROS: Minimal interaction rapid organ segmentation
RKV: Right kidney volume
TRUFI: True fast imaging with steady-state free precession
