# Supplementary material for: A rapid high-performance semi-automated tool to measure total kidney volume from MRI in autosomal dominant polycystic kidney disease
Source: Eur Radiol. 2019 Jan 21;29(8):4188–97. doi: 10.1007/s00330-018-5918-9 (PMC6610271; doi:10.1007/s00330-018-5918-9)

**Supplementary information**

**Table S1**: **Comparison of published MRI based methods to measure TKV in ADPKD patients.**

| Method | MRI sequence | Results | | | | Comments | Reference | |
| --- | --- | --- | --- | --- | --- | --- | --- | --- |
|  |  | No. of  patients | Time (min-secs) | Volume range (ml) | % volume  difference* |  |  |  |
| Stereology:  measuring TKV from  overlap of a grid and  kidney region on each slice | T2 FSE breath  hold | Phantom  + 4 | 30-60 s  per slice | 200-625 | - | Operator dependent, requires  specific computer software and  is less reliable in kidneys with a  few large cysts. | [10] |  |
| Ellipsoid:  equates kidney to an ellipsoid  (product of kidney length, width, depth) | - | 590 +  177  (external  validation) | 7 mins | (Ht TKV)  141-6167 | -0.6 ± 7.8 | Simple, not accurate - as kidney  approximated to ellipsoid,  no kidney region outline is  obtained, less accurate if eGFR>60ml/min. | [6] |  |
| Mid-Slice:  product of kidney area  on coronal mid-slice  by number of slices | T2 SS-FSE  fat saturation  (CRISP data) | 178 | - | - | R2: 0.994 LK  and 0.991 RK | Simple and fast, calculates  volume only, not accurate as  total volume based on single  slice area. | [22] |  |
| Seed-based threshold  segmentation with region  growing and morphological  operation | T2 TSE with selective fat  suppression  (CRISP data) | 30 | <2 mins | 693-2029 | -1.3 ± 3.9 | Volumes from coronal and axial plane, cannot segment detached cysts, may fail to detect kidney contour in presence of artefact. | [28] |  |
| Minimal interaction rapid  organ segmentation  (MIROS) | T2 SS-FSE | 40 | 5 mins | 661-2837 | -1.15 ± 7.84  -0.41 ± 8.19 | Semi-automatic, include renal  pelvis in segmentation outline,  minor inclusion of other organs. | [18] |  |
| Spatial prior probability  map and propagated  shape constraint into  level set framework | T2 SS-FSE  with fat  saturation  (CRISP data) | 60 (30  training +  30  validation) | - | 177-2634 | 4.2 ± 16.8 | Automatic technique, over –  estimates TKV, liver cysts  might be included in kidney  outline, needs good training set. | [12] |  |

CRISP, Consortium for Radiologic Imaging Studies of Polycystic Kidney Disease; Ht, Height adjusted; LK, left kidney; MRI, magnetic resonance imaging; RK, right kidney; R2, coefficient of determination; TKV, total kidney volume; SS-FSE, single-shot fast spin-echo (HASTE); TSE, turbo spin-echo; *% volume difference was calculated using manual measurement as reference.

**Figure S1 Examples of spatial overlap between Manual TKV (red) and *Sheffield TKV Tool* (green) for right and left kidneys.**

Each coronal slice is from a different patient. *Sheffield TKV Tool* can accurately segment kidney regions of different shape, size (a, b, c), cystic burden (g, h) or spatial position (f). *Sheffield TKV Tool* (green) may under-segment the region in exophytic kidney case (d) or over-segment in presence of liver cysts (i) and may include blood vessel (e, j) leading to large % volume difference compared to reference manual method.


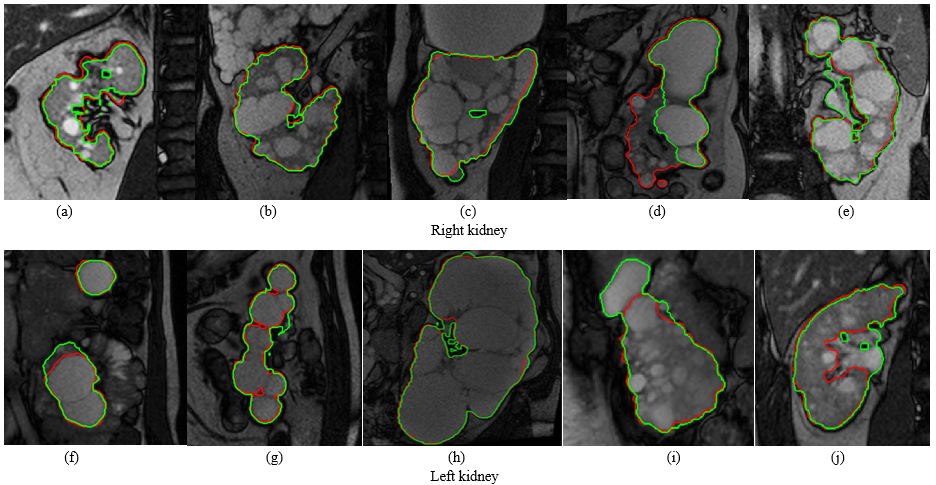

Supplement: Supplementary file 1 — (DOCX 529 kb) [file 330_2018_5918_MOESM1_ESM.docx]
